# 1-(4-Chloro­phen­yl)-3-(morpholin-4-yl)urea

**DOI:** 10.1107/S1600536812012615

**Published:** 2012-03-28

**Authors:** Yu-Feng Li, Jin-He Jiang

**Affiliations:** a Microscale Science Institute, Department of Chemistry and Chemical Engineering, Weifang University, Weifang 261061, People’s Republic of China

## Abstract

In the title mol­ecule, C_11_H_14_ClN_3_O_2_, the morpholine ring has a chair conformation. In the crystal, pairs of mol­ecules are linked into inversion dimers by N—H⋯O hydrogen bonds.

## Related literature

For the medicinal properties of related compounds, see: Yang *et al.* (1997 ▶). For a related structure, see: Li (2011 ▶).
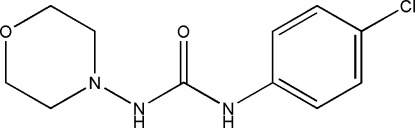


## Experimental

### 

#### Crystal data

C_11_H_14_ClN_3_O_2_
*M* *_r_* = 255.70
Monoclinic, 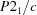

*a* = 13.684 (3) Å
*b* = 9.3612 (19) Å
*c* = 9.758 (2) Å
β = 94.03 (3)°
*V* = 1246.8 (4) Å^3^
*Z* = 4
Mo *K*α radiation
μ = 0.30 mm^−1^
*T* = 293 K
0.22 × 0.20 × 0.19 mm

#### Data collection

Bruker SMART CCD diffractometer
11894 measured reflections
2857 independent reflections
1502 reflections with *I* > 2σ(*I*)
*R*_int_ = 0.043

#### Refinement

*R*[*F* ^2^ > 2σ(*F* ^2^)] = 0.045
*wR*(*F* ^2^) = 0.180
*S* = 1.17
2857 reflections
154 parameters
H-atom parameters constrained
Δρ_max_ = 0.20 e Å^−3^
Δρ_min_ = −0.29 e Å^−3^

### 

Data collection: *SMART* (Bruker 1997 ▶); cell refinement: *SAINT* (Bruker 1997 ▶); data reduction: *SAINT*; program(s) used to solve structure: *SHELXS97* (Sheldrick, 2008 ▶); program(s) used to refine structure: *SHELXL97* (Sheldrick, 2008 ▶); molecular graphics: *SHELXTL* (Sheldrick, 2008 ▶); software used to prepare material for publication: *SHELXTL*.

## Supplementary Material

Crystal structure: contains datablock(s) global, I. DOI: 10.1107/S1600536812012615/lh5438sup1.cif

Structure factors: contains datablock(s) I. DOI: 10.1107/S1600536812012615/lh5438Isup2.hkl

Supplementary material file. DOI: 10.1107/S1600536812012615/lh5438Isup3.cml

Additional supplementary materials:  crystallographic information; 3D view; checkCIF report

## Figures and Tables

**Table 1:** Hydrogen-bond geometry (Å, °)

*D*—H⋯*A*	*D*—H	H⋯*A*	*D*⋯*A*	*D*—H⋯*A*
N2—H2*A*⋯O2^i^	0.86	2.03	2.865 (3)	162

Symmetry code: (i) 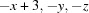.

**Fig. 1.**
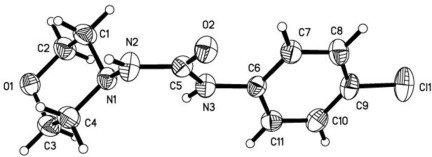
The structure of the title compound showing 30% probability displacement ellipsoids.

## supplementary crystallographic information

### Comment

Some compounds which are related to the title compound have been shown to have medicinal properties (Yang *et al.*, 1997). The molecular structure of the title compound is shown in Fig .1. The morpholine ring (C1-C4/O1/N1) has a chair conformation. The bond lengths and angles can be compared to those within a related structure (Li, 2011). In the crystal, pairs of molecules are linked into inversion dimers by N—H···O hydrogen bonds.

### Experimental

A mixture of 4-aminomorpholine (0.08 mol), and (4-chlorophenyl)carbamic chloride (0.08 mol) was stirred in refluxing ethanol (18 ml) for 4 h to afford the title compound (0.064 mol, yield 80%). Colourless blocks of the title compound were obtained by recrystallization from ethanol at room temperature.

### Refinement

H atoms were fixed geometrically and allowed to ride on their attached atoms, with C—H distances = 0.93–0.97 Å; N—H = 0.86 Å and with *U*_iso_(H) = 1.2*U*_eq_(C,*N*).

### Crystal data

**Table:**

C_11_H_14_ClN_3_O_2_	*F*(000) = 536
*M**_r_* = 255.70	*D*_x_ = 1.362 Mg m^−^^3^
Monoclinic, *P*2_1_/*c*	Mo *K*α radiation, λ = 0.71073 Å
Hall symbol: -P 2ybc	Cell parameters from 1502 reflections
*a* = 13.684 (3) Å	θ = 3.0–27.5°
*b* = 9.3612 (19) Å	µ = 0.30 mm^−^^1^
*c* = 9.758 (2) Å	*T* = 293 K
β = 94.03 (3)°	Block, colorless
*V* = 1246.8 (4) Å^3^	0.22 × 0.20 × 0.19 mm
*Z* = 4	

### Data collection

**Table:**

Bruker SMART CCD diffractometer	1502 reflections with *I* > 2σ(*I*)
Radiation source: fine-focus sealed tube	*R*_int_ = 0.043
Graphite monochromator	θ_max_ = 27.5°, θ_min_ = 3.0°
φ and ω scans	*h* = −17→17
11894 measured reflections	*k* = −12→12
2857 independent reflections	*l* = −12→12

### Refinement

**Table:**

Refinement on *F*^2^	Primary atom site location: structure-invariant direct methods
Least-squares matrix: full	Secondary atom site location: difference Fourier map
*R*[*F*^2^ > 2σ(*F*^2^)] = 0.045	Hydrogen site location: inferred from neighbouring sites
*wR*(*F*^2^) = 0.180	H-atom parameters constrained
*S* = 1.17	*w* = 1/[σ^2^(*F*_o_^2^) + (0.085*P*)^2^ + 0.0207*P*] where *P* = (*F*_o_^2^ + 2*F*_c_^2^)/3
2857 reflections	(Δ/σ)_max_ < 0.001
154 parameters	Δρ_max_ = 0.20 e Å^−^^3^
0 restraints	Δρ_min_ = −0.29 e Å^−^^3^

### Special details

**Table:**

Geometry. All e.s.d.'s (except the e.s.d. in the dihedral angle between two l.s. planes) are estimated using the full covariance matrix. The cell e.s.d.'s are taken into account individually in the estimation of e.s.d.'s in distances, angles and torsion angles; correlations between e.s.d.'s in cell parameters are only used when they are defined by crystal symmetry. An approximate (isotropic) treatment of cell e.s.d.'s is used for estimating e.s.d.'s involving l.s. planes.
Refinement. Refinement of *F*^2^ against ALL reflections. The weighted *R*-factor *wR* and goodness of fit *S* are based on *F*^2^, conventional *R*-factors *R* are based on *F*, with *F* set to zero for negative *F*^2^. The threshold expression of *F*^2^ > σ(*F*^2^) is used only for calculating *R*-factors(gt) *etc*. and is not relevant to the choice of reflections for refinement. *R*-factors based on *F*^2^ are statistically about twice as large as those based on *F*, and *R*- factors based on ALL data will be even larger.

### Fractional atomic coordinates and isotropic or equivalent isotropic displacement parameters (Å^2^)

**Table:**

	*x*	*y*	*z*	*U*_iso_*/*U*_eq_	
Cl1	0.92775 (5)	0.08842 (12)	0.30739 (8)	0.0988 (4)	
O2	1.39233 (12)	0.0438 (2)	0.09026 (16)	0.0612 (5)	
C6	1.18312 (16)	0.0784 (3)	0.0412 (2)	0.0493 (6)	
N3	1.25450 (14)	0.0646 (2)	−0.0549 (2)	0.0561 (5)	
H3A	1.2345	0.0708	−0.1403	0.067*	
N1	1.35490 (13)	0.0024 (2)	−0.26930 (19)	0.0535 (5)	
O1	1.35069 (15)	−0.0687 (2)	−0.55127 (17)	0.0697 (5)	
N2	1.40369 (14)	0.0221 (3)	−0.1380 (2)	0.0656 (6)	
H2A	1.4667	0.0212	−0.1292	0.079*	
C5	1.35207 (17)	0.0425 (3)	−0.0260 (2)	0.0504 (6)	
C8	1.1131 (2)	0.0212 (3)	0.2519 (3)	0.0674 (7)	
H8A	1.1185	−0.0217	0.3381	0.081*	
C11	1.09794 (16)	0.1525 (3)	−0.0004 (3)	0.0592 (6)	
H11A	1.0931	0.1986	−0.0850	0.071*	
C9	1.02870 (18)	0.0905 (3)	0.2072 (3)	0.0644 (7)	
C10	1.02095 (18)	0.1582 (3)	0.0826 (3)	0.0672 (7)	
H10A	0.9641	0.2076	0.0543	0.081*	
C7	1.19009 (18)	0.0152 (3)	0.1684 (2)	0.0608 (7)	
H7A	1.2474	−0.0321	0.1986	0.073*	
C3	1.3409 (2)	0.0735 (3)	−0.5071 (3)	0.0734 (8)	
H3B	1.2720	0.0954	−0.5020	0.088*	
H3C	1.3669	0.1373	−0.5739	0.088*	
C4	1.3938 (2)	0.0989 (3)	−0.3690 (3)	0.0666 (7)	
H4A	1.4634	0.0822	−0.3741	0.080*	
H4B	1.3847	0.1972	−0.3409	0.080*	
C2	1.3146 (2)	−0.1633 (3)	−0.4542 (2)	0.0763 (8)	
H2B	1.3226	−0.2609	−0.4849	0.092*	
H2C	1.2451	−0.1462	−0.4478	0.092*	
C1	1.3671 (2)	−0.1449 (3)	−0.3145 (3)	0.0709 (8)	
H1A	1.3404	−0.2101	−0.2496	0.085*	
H1B	1.4362	−0.1662	−0.3191	0.085*	

### Atomic displacement parameters (Å^2^)

**Table:**

	*U*^11^	*U*^22^	*U*^33^	*U*^12^	*U*^13^	*U*^23^
Cl1	0.0664 (5)	0.1530 (10)	0.0797 (6)	−0.0135 (5)	0.0244 (4)	−0.0336 (5)
O2	0.0541 (9)	0.0801 (13)	0.0483 (10)	0.0043 (8)	−0.0044 (8)	−0.0082 (8)
C6	0.0484 (12)	0.0545 (15)	0.0446 (12)	0.0017 (10)	0.0000 (10)	−0.0067 (10)
N3	0.0462 (10)	0.0747 (15)	0.0468 (11)	0.0059 (9)	−0.0007 (8)	−0.0015 (9)
N1	0.0532 (11)	0.0632 (14)	0.0438 (10)	0.0018 (9)	0.0020 (9)	−0.0021 (9)
O1	0.0918 (13)	0.0737 (13)	0.0449 (9)	0.0106 (10)	0.0143 (9)	0.0027 (9)
N2	0.0474 (11)	0.0996 (19)	0.0493 (11)	0.0054 (11)	−0.0004 (9)	−0.0114 (11)
C5	0.0500 (12)	0.0519 (15)	0.0488 (13)	0.0021 (10)	0.0005 (10)	−0.0025 (10)
C8	0.0644 (15)	0.090 (2)	0.0474 (13)	−0.0009 (15)	0.0025 (12)	−0.0051 (13)
C11	0.0540 (13)	0.0626 (17)	0.0605 (14)	0.0053 (12)	0.0002 (11)	0.0021 (13)
C9	0.0565 (14)	0.080 (2)	0.0576 (15)	−0.0081 (13)	0.0085 (12)	−0.0203 (14)
C10	0.0518 (13)	0.073 (2)	0.0767 (18)	0.0061 (13)	0.0022 (13)	−0.0102 (15)
C7	0.0557 (13)	0.0742 (19)	0.0519 (14)	0.0092 (13)	0.0002 (11)	−0.0006 (13)
C3	0.093 (2)	0.069 (2)	0.0590 (16)	0.0070 (16)	0.0062 (14)	0.0129 (14)
C4	0.0748 (17)	0.0613 (18)	0.0641 (16)	−0.0030 (13)	0.0083 (13)	0.0052 (13)
C2	0.111 (2)	0.0663 (19)	0.0530 (15)	−0.0077 (17)	0.0139 (15)	−0.0052 (13)
C1	0.103 (2)	0.0593 (17)	0.0501 (13)	0.0035 (16)	0.0059 (14)	0.0055 (12)

### Geometric parameters (Å, º)

**Table:**

Cl1—C9	1.748 (3)	C8—H8A	0.9300
O2—C5	1.226 (3)	C11—C10	1.374 (3)
C6—C7	1.373 (3)	C11—H11A	0.9300
C6—C11	1.392 (3)	C9—C10	1.369 (4)
C6—N3	1.407 (3)	C10—H10A	0.9300
N3—C5	1.361 (3)	C7—H7A	0.9300
N3—H3A	0.8600	C3—C4	1.503 (4)
N1—N2	1.414 (3)	C3—H3B	0.9700
N1—C4	1.456 (3)	C3—H3C	0.9700
N1—C1	1.461 (3)	C4—H4A	0.9700
O1—C3	1.409 (4)	C4—H4B	0.9700
O1—C2	1.411 (3)	C2—C1	1.505 (4)
N2—C5	1.356 (3)	C2—H2B	0.9700
N2—H2A	0.8600	C2—H2C	0.9700
C8—C9	1.369 (4)	C1—H1A	0.9700
C8—C7	1.377 (4)	C1—H1B	0.9700
			
C7—C6—C11	118.8 (2)	C6—C7—C8	120.7 (2)
C7—C6—N3	123.8 (2)	C6—C7—H7A	119.7
C11—C6—N3	117.3 (2)	C8—C7—H7A	119.7
C5—N3—C6	126.35 (19)	O1—C3—C4	112.0 (2)
C5—N3—H3A	116.8	O1—C3—H3B	109.2
C6—N3—H3A	116.8	C4—C3—H3B	109.2
N2—N1—C4	110.6 (2)	O1—C3—H3C	109.2
N2—N1—C1	109.9 (2)	C4—C3—H3C	109.2
C4—N1—C1	109.2 (2)	H3B—C3—H3C	107.9
C3—O1—C2	110.0 (2)	N1—C4—C3	109.0 (2)
C5—N2—N1	120.60 (19)	N1—C4—H4A	109.9
C5—N2—H2A	119.7	C3—C4—H4A	109.9
N1—N2—H2A	119.7	N1—C4—H4B	109.9
O2—C5—N2	121.4 (2)	C3—C4—H4B	109.9
O2—C5—N3	124.1 (2)	H4A—C4—H4B	108.3
N2—C5—N3	114.4 (2)	O1—C2—C1	111.6 (2)
C9—C8—C7	119.6 (3)	O1—C2—H2B	109.3
C9—C8—H8A	120.2	C1—C2—H2B	109.3
C7—C8—H8A	120.2	O1—C2—H2C	109.3
C10—C11—C6	120.6 (2)	C1—C2—H2C	109.3
C10—C11—H11A	119.7	H2B—C2—H2C	108.0
C6—C11—H11A	119.7	N1—C1—C2	108.9 (2)
C8—C9—C10	120.9 (2)	N1—C1—H1A	109.9
C8—C9—Cl1	119.9 (2)	C2—C1—H1A	109.9
C10—C9—Cl1	119.1 (2)	N1—C1—H1B	109.9
C9—C10—C11	119.4 (2)	C2—C1—H1B	109.9
C9—C10—H10A	120.3	H1A—C1—H1B	108.3
C11—C10—H10A	120.3		

### Hydrogen-bond geometry (Å, º)

**Table:**

*D*—H···*A*	*D*—H	H···*A*	*D*···*A*	*D*—H···*A*
N2—H2*A*···O2^i^	0.86	2.03	2.865 (3)	162

Symmetry code: (i) −*x*+3, −*y*, −*z*.
